# Cytoprotective Effect of the Elongation Factor-2 Kinase-Mediated Autophagy in Breast Cancer Cells Subjected to Growth Factor Inhibition

**DOI:** 10.1371/journal.pone.0009715

**Published:** 2010-03-16

**Authors:** Yan Cheng, Huaijun Li, Xingcong Ren, Tingkuang Niu, William N. Hait, Jinming Yang

**Affiliations:** 1 Department of Pharmacology and The Penn State Cancer Institute, The Pennsylvania State University College of Medicine, and Milton S. Hershey Medical Center, Hershey, Pennsylvania, United States of America; 2 Department of Pharmacology, School of Medicine, Soochow University, Suzhou, China; 3 OrthoBiotech Research and Development, Raritan, New Jersey, United States of America; Roswell Park Cancer Institute, United States of America

## Abstract

**Background:**

Autophagy is a highly conserved and regulated cellular process employed by living cells to degrade proteins and organelles as a response to metabolic stress. We have previously reported that eukaryotic elongation factor-2 kinase (eEF-2 kinase, also known as Ca^2+^/calmodulin-dependent protein kinase III) can positively modulate autophagy and negatively regulate protein synthesis. The purpose of the current study was to determine the role of the eEF-2 kinase-regulated autophagy in the response of breast cancer cells to inhibitors of growth factor signaling.

**Methodology/Principal Findings:**

We found that nutrient depletion or growth factor inhibitors activated autophagy in human breast cancer cells, and the increased activity of autophagy was associated with a decrease in cellular ATP and an increase in activities of AMP kinase and eEF-2 kinase. Silencing of eEF-2 kinase relieved the inhibition of protein synthesis, led to a greater reduction of cellular ATP, and blunted autophagic response. We further showed that suppression of eEF-2 kinase-regulated autophagy impeded cell growth in serum/nutrient-deprived cultures and handicapped cell survival, and enhanced the efficacy of the growth factor inhibitors such as trastuzumab, gefitinib, and lapatinib.

**Conclusion/Significance:**

The results of this study provide new evidence that activation of eEF-2 kinase-mediated autophagy plays a protective role for cancer cells under metabolic stress conditions, and that targeting autophagic survival may represent a novel approach to enhancing the effectiveness of growth factor inhibitors.

## Introduction

Autophagy is a highly conserved process by which cytoplasm and organelles are digested via autophagosomes and autolysosomes and cellular components are recycled for energy utilization [1], [2]. During starvation or growth factor deficiency, autophagy may serve as a temporary survival mechanism by providing an alternative energy source. Autophagy can also optimize nutrient utilization in rapidly growing cells when faced with hypoxic or metabolic stresses, thus contributing to cancer cell survival [3], [4], [5]. eEF-2 kinase, a Ca^2+^/calmodulin-dependent protein kinase, acts as a negative regulator of protein synthesis: this kinase phosphorylates eEF-2, a 100 kDa protein that mediates the translocation step in peptide-chain elongation by inducing the transfer of peptidyl-tRNA from the ribosomal A to P site; phosphorylation of eEF-2 at Thr56 by eEF-2 kinase decreases the affinity of the elongation factor for ribosome and terminates elongation [6]. Our previous studies demonstrated that eEF-2 kinase might be a central component of the mammalian macroautophagy pathway that is activated in response to nutrient deprivation [7], [8]. The role of eEF-2 kinase in the regulation of stress-induced autophagy has further been confirmed by others [9]. Since protein synthesis is a major energy-consuming process, termination of protein synthesis and induction of autophagy via activation of eEF-2 kinase should conserve energy and support cell survival during time of metabolic stress. Moreover, eEF-2 kinase has been found to be overexpressed and its activity increased in multiple breast cancer cell lines and human breast cancer specimens as compared to adjacent normal tissue [10].

The members of the epidermal growth factor receptor (EGFR) family such as EGFR/HER1 and HER2/erB2 represent attractive targets for therapeutic intervention in treatment of cancer, due to the roles of these receptor tyrosine kinases in stimulating oncogenic signaling pathways and in the development and progression of cancers [11], [12], [13]. Aberrant expression or activity of the EGFR family receptor tyrosine kinases is encountered in many types of malignancies including breast cancers. Indeed, the EGFR tyrosine kinase inhibitors such as lapatinib and gefitinib, and the HER2/neu-targeted agent trastuzumab, have been shown to possess notable antitumor activity in several types of cancers [14]. These drugs can specifically bind to the receptors with high affinity, resulting in blockade of the downstream signaling pathways and inhibition of tumor growth. Nevertheless, refractoriness to these growth factor inhibitors is common [15], [16]. For instance, in patients with HER2-positive metastatic breast cancers, the response rate of trastuzumab is only ∼26% [17]. Thus, understanding of the mechanisms underlying the insensitivity to the growth factors inhibitors and developing approaches to sensitizing tumor cells will make these drugs more valuable in treating patients with cancer. In this study, we sought to determine whether activation of eEF-2 kinase-mediated autophagy altered sensitivity of human breast cancer cells to inhibition of growth factor-initiated signaling, and whether modulating autophagy via targeting eEF-2 kinase would render tumor cells more susceptible to the effect of growth factor inhibitors. Our study shows that the eEF-2 kinase-mediated autophagy plays a cytoprotective role in breast cancer cells treated with growth factor inhibitors, and inhibiting autophagic survival can modulate sensitivity to these therapeutic agents.

## Results

### Nutrient deprivation and growth factor inhibitors activated autophagy in human breast cancer cells

**Figure 1:**
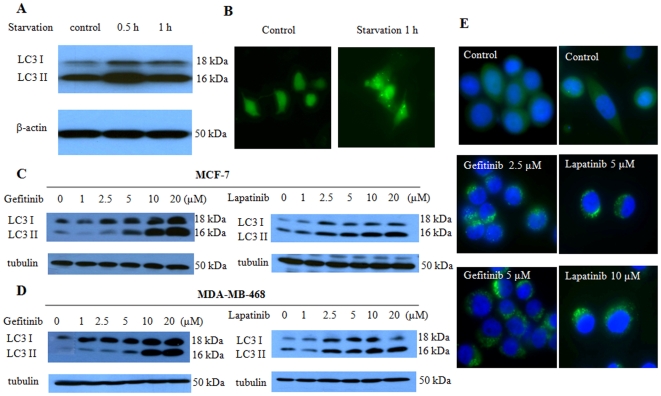
Effect of nutrient deprivation and growth factor inhibitors on autophagy in human breast cancer cells. (**A**) MCF-7 cells were treated with DPBS for the indicated times. At the end of treatment, formation of the autophagy marker LC3-II was detected by immunoblotting with an anti-MAP-LC3 antibody. (**B**) MCF-7 cells transfected with 3 µg of GFP-LC3 plasmid were treated or untreated with DPBS for 1 h, and then observed under a fluorescent microscope. A representation of GFP-LC3 positive cells was shown. (**C, D**) MCF-7 (C) or MDA-MB-468 (D) cells cultured in medium containing 10% FBS were treated with 0.5, 1, 2.5, 5 or 10 µM gefitinib or lapatinib for 24 h, and the LC3-II level was examined by immunoblotting. (**E**) MDA-MB-468 cells were transfected with a GFP-LC3-expressing vector, and then treated with the indicated concentration of gefitinib or lapatinib in the presence of the lysosomal protease inhibitors E64d (10 µg/ml) and pepstain A (10 µg/ml). At the end of treatments, cells were fixed with 4% formaldehyde for 15 min. To determine the autophagic response, cells were inspected at 60× magnification for numbers of GFP-LC3 puncta.

To determine the effect of metabolic stress on autophagy, we first treated the human breast cancer cells MCF-7 with DPBS, and then examined autophagic activity in the treated cells. As shown in Fig. 1A, nutrient deprivation increased the level of LC3-II, a specific marker of autophagic activity. Autophagosome formation was confirmed by GFP-LC3 puncta localization (Fig. 1B). We further found that MCF-7 and MDA-MB-468 breast cancer cells treated with the growth factor inhibitors, gefitinib and lapatinib, showed an increased in LC3-II amount in a dose-dependent manner, as determined by Western blot (Fig. 1C and D). The effects of gefitinib and lapatinib on autophagy were verified using GFP-LC3 cleavage assay (Fig. 1E). These observations suggest that activation of autophagy may represent a cellular response to metabolic stress, including treatment with growth factor inhibitors.

### Nutrient deprivation caused a reduction of protein synthesis and cellular ATP

**Figure 2:**
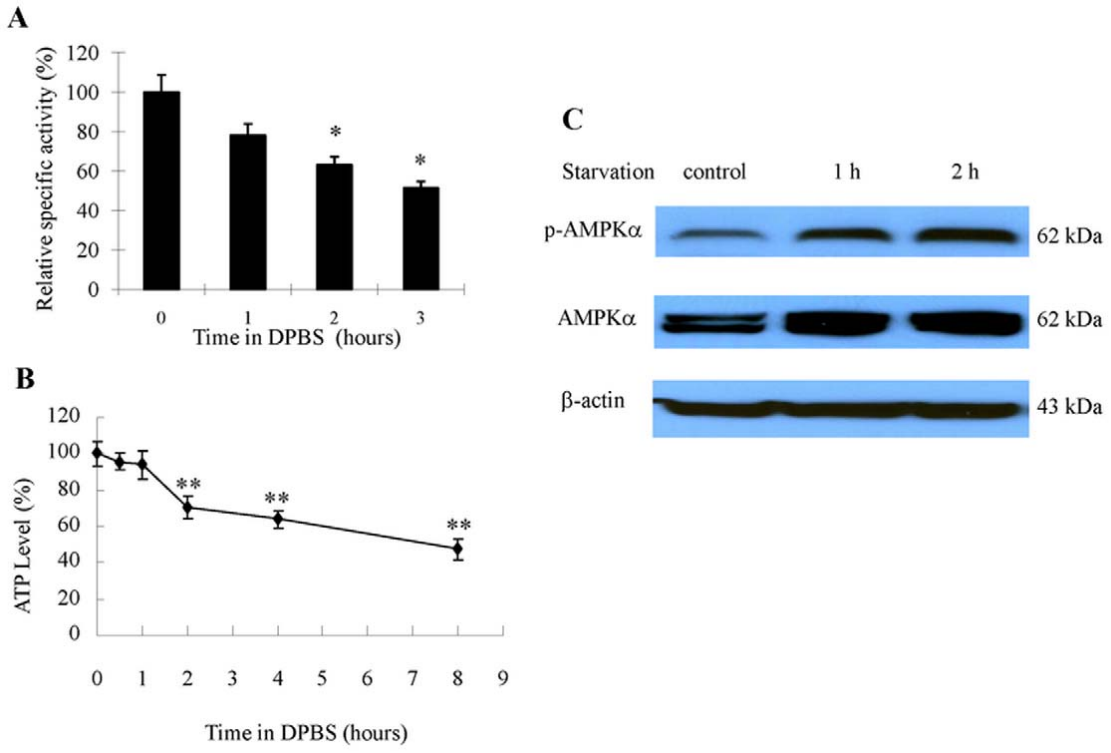
Effect of nutrient deprivation on protein synthesis and cellular ATP in MCF-7 cells. (**A**) MCF-7 cells were treated with DPBS for the indicated times and the rate of protein synthesis was measured by labeling the cells with 25 µCi/ml of EasyTag EXPRESS [^35^S] protein labeling mix and liquid scintillation counting, as described in “Material and Methods”. The specific activity of protein synthesis was determined by the amount of incorporated ^35^S-methionine/cysteine per mg of total protein per min, and relative activities at indicated times of starvation were calculated as percent of control. Results shown are the mean ± SD of quadruplicate determinations from one of three identical experiments; *p<0.05, *t*-test. (**B**) MCF-7 cells were treated with DPBS for the indicated times and ATP content was measured using the ATPlite™ Luminescence Assay Kit. (**C**) AMPK activity was determined by Western blot analysis of phospho-AMPK using an anti-phospho-AMPK antibody, as described in “Material and Methods”. Actin was used as a loading control. Results shown are the representative of three similar experiments; each bar or point represents mean ± SD of quadruplicate determinations. Results shown are the mean ± SD of quadruplicate determinations from one of three identical experiments; **p<0.01, *t*-test.

To ascertain whether a causal relationship exists between activation of autophagy and metabolic stress in breast cancer cells, we measured protein synthesis activity and ATP level in MCF-7 cells subjected to nutrient starvation. As shown in Fig. 2, treatment of the cells with DPBS (Dulbecco's Phosphate-Buffered Saline) caused a marked decrease in protein synthesis (Fig. 2A) and ATP content (Fig. 2B). Cellular response to energy stress was also evidenced by an increased phosphorylation on Thr172 of AMP-activated protein kinase (AMPK), an intracellular energy sensor that is activated when cellular energy level decreases (Fig. 2C).

### Nutrient deprivation activated eEF-2 kinase through the mTOR/S6 kinase pathway

**Figure 3:**
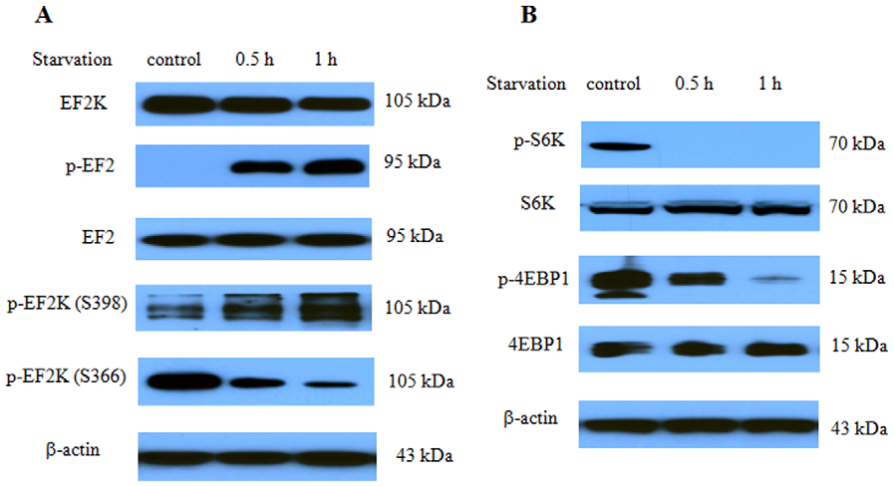
Effect of nutrient deprivation on eEF-2 kinase activity and the associated signaling molecules. (**A**) MCF-7 cells were treated with DPBS for the indicated times, and the levels of eEF-2 kinase, p-EF2, EF2, p-eEF2 kinase (S398) and p-eEF2 kinase (S366) were examined by Western blot using the respective antibodies. (**B**) MCF-7 cells were treated with DPBS for the indicated times, and p-S6 kinase, S6 kinase, p-4EBP1, and 4EBP1 were examined by Western blot using the respective antibodies. β-actin was used as a loading control. Results shown are the representative of three similar experiments.

We next determined the effects of nutrient deprivation on the activity of eEF-2 kinase, a unique calmodulin-dependent enzyme that inhibits protein synthesis and activates autophagy, and on the signaling molecules associated with the regulation of the activity of this kinase. Fig. 3A shows that treatment of MCF-7 cells with DPBS activated eEF-2 kinase, as measured by the phosphorylation of EF-2, the substrate for the kinase. Activation of eEF-2 kinase was also manifested in an increased auto-phosphorylation on Ser398, which is known to positively regulate the activity of this kinase, and in a decreased phosphorylation of the kinase on Ser366, a site known to negatively regulate the activity of this enzyme (Fig. 3A). The activity of S6 kinase, a key translation controller downstream of mTOR, was decreased, as evidenced by a decrease in the phosphorylation on Thr389 of S6 kinase (Fig. 3B). The activity of 4EBP1, a translation repressor, was increased, as shown by a decrease in the phosphorylation of this protein (Fig. 3B).

### eEF-2 kinase was involved in autophagy induction and ATP reduction in response to nutrient depletion

**Figure 4:**
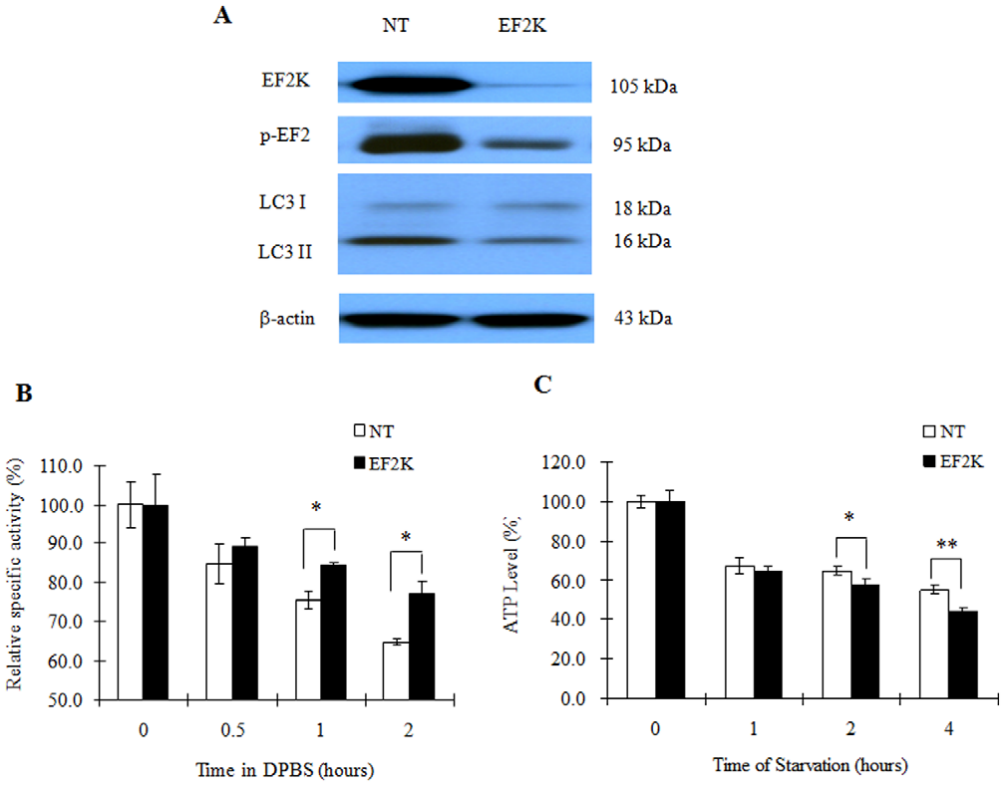
Inhibition of eEF-2 kinase blunts autophagy, mitigates inhibition of protein synthesis and hastens reduction of cellular ATP. (**A**) MCF-7 cells were transfected with a non-targeting RNA or an eEF-2 kinase-targeted siRNA (100 nM) for 72 h, and then treated with DPBS for 1 h. eEF-2 kinase, phosphor-EF-2, and the autophagy marker, LC3-II, were detected by Western blot. (**B**) MCF-7 cells with or without silencing of eEF-2 kinase were treated with DPBS; at the indicated times, cells were harvested for protein synthesis assay. Results shown are the mean ± SD of quadruplicate determinations from one of three identical experiments; *p<0.05, *t*-test. (**C**) MCF-7 cells transfected with 50 nM of NT RNA or an eEF-2 kinase siRNA were seeded in 96-well tissue culture plates (1×10^4^ cells per well). Forty-eight h later, the cells were starved in DPBS for 1 h, 2 h and 4 h. Cells were collected at the end of starvation for ATP assay. Results shown are the mean ± SD of quadruplicate determinations from one of three identical experiments; *p<0.05, **p<0.01, *t*-test.

To analyze whether eEF-2 kinase plays a regulatory role in nutrient starvation-induced reduction of protein synthesis and ATP content and in activation of autophagy in breast cancer cells stressed with nutrient depletion, we silenced EF-2 kinase expression using siRNA, and then measured ATP level, protein synthesis and autophagy activity following treatment with DPBS. As shown in Fig. 4A, inhibition of eEF-2 kinase by siRNA decreased starvation-induced autophagy. Inhibition of eEF-2 kinase also resulted in mitigation of the nutrient depletion-induced inhibition of protein synthesis (Fig. 4B) and led to further reduction of ATP levels (Fig. 4C). These results further support a role for eEF-2 kinase in activating autophagy in metabolically stressed tumor cells.

### Suppression of autophagy decreased growth and survival of metabolically stressed breast cancer cells

**Figure 5:**
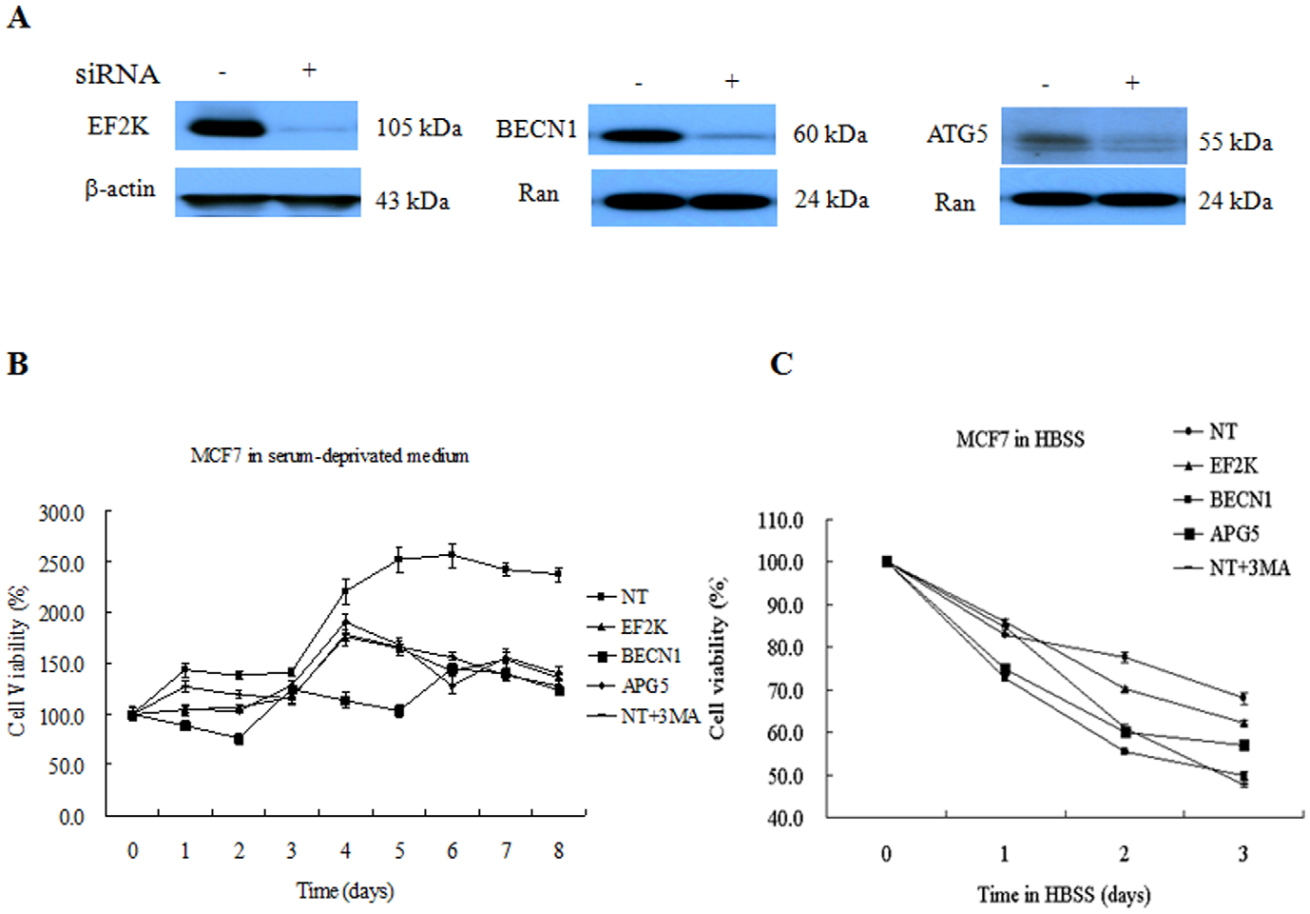
Effects of autophagy suppression on growth and survival of human breast cancer cells. (**A**) MCF-7 cells were transfected with a non-targeting RNA or a siRNA targeting eEF-2 kinase, beclin1, or ATG5. Expressions of eEF-2 kinase, beclin1, or ATG5 were determined by Western blot using the respective antibodies. β-actin or Ran was used as a loading control. (**B**) MCF-7 cells treated with 3-MA or with siRNA targeting eEF-2 kinase, beclin1, ATG5 or a non-targeting RNA were seeded with 10% FBS RPMI 1640 medium in 96-well culture plates (3×10^3^ cells per well). After overnight incubation, medium was changed to serum-free medium. Cell viability was determined at the indicated times using MTT assay. Results shown are the representative of three similar experiments; each point represents mean ± SD of quadruplicate determinations. (**C**) MCF-7 cells treated with 3-MA or with siRNA targeting eEF-2 kinase, beclin1, ATG5 or a non-targeting RNA were seeded with 10% FBS RPMI 1640 medium in 96-well culture plates (3×10^3^ cells per well). After overnight incubation, medium was changed to HBSS. Cell viability was determined at the indicated times using MTT assay. Results shown are the representative of three similar experiments; each point represents mean ± SD of quadruplicate determinations.

To further test our hypothesis that autophagy plays a pro-survival role in response to a compromised supply of cellular nutrients and growth factors during breast cancer development and progression, we knocked down eEF-2 kinase, beclin-1 or ATG5 (two of the key autophagy-related genes) in MCF-7 cells (Fig. 5A), and then compared the growth and survival of these autophagy-deficient cells with that of the cells transfected with a non-targeting RNA in serum-free medium or HBSS. As shown in Fig. 5B, suppression of autophagy by knockdown of those autophagy-regulated genes hindered the tumor cell growth in the absence of serum. Knockdown of eEF-2 kinase, beclin-1 or ATG5 also caused more death of MCF-7 cells cultured in HBSS (Fig. 5C). The autophagy inhibitor, 3-MA, was used as a control and showed a similar inhibitory effect on cell growth and survival in the absence of serum or nutrients (Fig. 5B and C).

### Inhibition of eEF-2 kinase sensitized breast cancer cells to growth factor inhibitors

**Figure 6:**
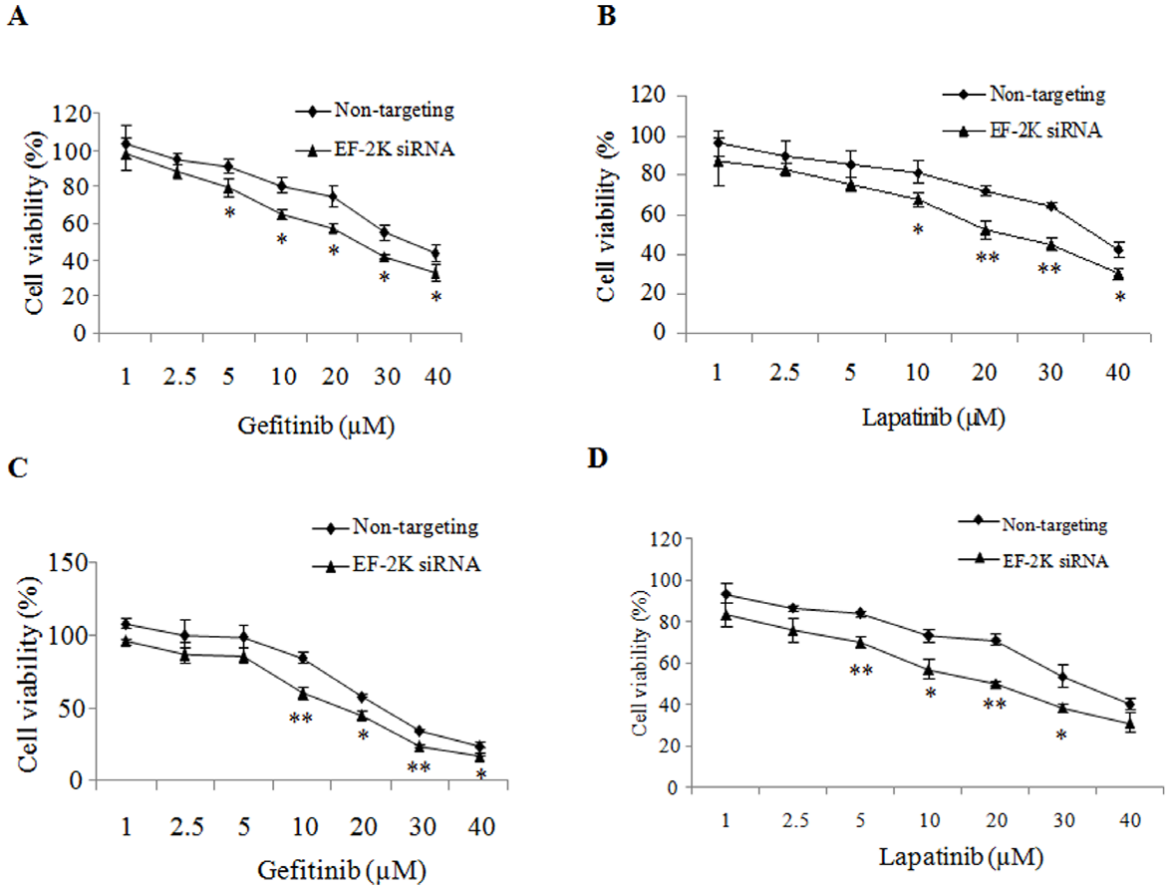
Effects of eEF-2 kinase silencing on sensitivity of human breast cancer cells to growth factor inhibitors. MCF-7 (**A, B**) and MDA-MB-468 (**C, D**) cells transfected with an siRNA targeting eEF-2 kinase or a non-targeting RNA were cultured in RPMI 1640 or DMEM media supplemented with 10% fetal bovine serum at 37°C in a humidified incubator (5% CO_2_ and 95% air), and then treated with a series of concentrations of gefitinib (**A, C**) or lapatinib (**B, D**) for 48 h. At the end of treatment, cell viability was determined using MTT assay. Results shown are the representative of three similar experiments; each point represents mean ± SD of quadruplicate determinations; *p<0.05, **p<0.01, *t*-test.

To determine whether suppression of the eEF-2 kinase-mediated autophagy alters sensitivity of tumor cells to growth factor inhibitors that are in clinical use, we first transfected MCF-7 cells with an eEF-2 kinase-targeted siRNA or a non-targeting RNA, and then treated the transfected cells with a series of concentrations of gefitinib or lapatinib. Fig. 6A and B show that silencing of EF-2 kinase expression increased sensitivity of MCF-7 cells to gefitinib and lapatinib. Similar results were observed with the human breast cancer cells MDA-MB-468 (Fig. 6C and D). Inhibition of autophagy by an eEF-2 kinase-targeted siRNA also enhanced the cytotoxic effects of a small molecule EGFR/ErbB-2 inhibitor (EEi) and trastuzumab, an anti-Her2 therapeutic antibody (Table 1). Combined use of the inhibitors of growth factor and autophagy produced combination indexes (CIs) smaller than 1 (Table 1), indicating a synergism between the actions of those treatments.

**Table 1:** CompuSyn analysis of the combinations of growth factor inhibitors and eEF-2 kinase siRNA.

Cell types	Samples	CI values
**MCF7**	Gefitinib: eEF-2K siRNA	0.45
	Lapatinib: eEF-2K siRNA	0.42
	EEi: eEF-2K siRNA	0.51
	Trastuzumab: eEF-2K siRNA	0.60
**MDA-MB-468**	Gefitinib: eEF-2K siRNA	0.40
	Lapatinib: eEF-2K siRNA	0.53
	EEi: eEF-2K siRNA	0.65
	Trastuzumab: eEF-2K siRNA	0.67

Combination index (CI) was calculated using the computer program, CompuSyn.

## Discussion

A number of molecular mechanisms have been revealed to contribute to resistance of cancer to drugs that target growth factor receptor-initiated signaling pathways. These known mechanisms include overexpression or mutation of EGFR [18], co-activation of multiple receptor tyrosine kinases [19], [20], mutation or alteration in the downstream effectors of EGFR such as loss of PTEN [21], and compensatory pathways that remain active in stimulating PI-3 kinase [22]. Here, we identified the activation of the eEF-2 kinase-mediated autophagy as a new mechanism responsible for the insensitivity of cancer cells to growth factor inhibition. We found that breast cancer cells utilize autophagy to survive nutrient deficiency and growth factor inhibitors, and eEF-2 kinase also plays an important role in the induction of autophagy triggered by growth factor inhibitors. We demonstrate that inhibitors of growth factors such as gefitinib and lapatinib can induce autophagy in breast cancer cells (Fig. 1), and the nutrient depletion-induced autophagy is associated with activation of eEF-2 kinase via the mTOR/S6 kinase signaling pathways (Fig. 3). Furthermore, we show that inhibiting the eEF-2 kinase-mediated autophagy can sensitize breast cancer cells to growth factor inhibitors (Fig. 6, Table 1). The results of the current study are consistent with our previous findings that eEF-2 kinase plays a critical role in autophagic survival of stressed tumor cells [7], [8]. A recent report by Vazquez-Martin et al. shows that autophagy also plays an essential role in the development of resistance to trastuzumab, an anti-Her2 therapeutic antibody, in breast cancer exposed chronically to this agent [23].

A variety of anticancer therapies induce autophagy, including antiestrogens [24], radiation [25], inhibitors of histone deacetylases [26] and chemotherapy [27], [28]. Whether the autophagic response is a pathway to cell death or a mechanism of survival (or both) remains uncertain. For instance, vinblastine increased the number of autophagosomes in pre-neoplastic pancreatic acini [29], whereas pancreatic cancer cells had a decreased autophagic response to vinblastine [30]. Radiation increased autophagy in glioma cell lines, but whether this was a survival or death response remains unclear, since the autophagic changes were independent of sensitivity to radiation [31]. More recently, it was reported that blocking autophagy could enhance the sensitivity of breast cancer to radiation [25]. It is likely that the pro-survival or pro-death role of autophagy is context-dependent.

Several points during breast cancer progression are characterized by a compromised supply of cellular nutrients and growth factors. For instance, when transformed cells invade the stroma or land in a metastatic site, these cells are temporarily “cut off” from a robust blood supply, decreasing access to oxygen, estrogen, glucose, amino acids, and other growth factors. To survive this nutrient-depleted state, breast cancer cells (much like neonates) could utilize autophagy to generate ATP from recycled organelles and long-lived proteins. To avert cell death, autophagic cells must limit protein synthesis to prevent the critical depletion of ATP. This is accomplished through inhibition of eIF4 and activation of eEF2-kinase to inhibit protein synthesis. Indeed, our studies demonstrated that failure to activate eEF-2 kinase blocked autophagy (Fig. 4), handicapped cell growth (Fig. 5B) and hastened cell death (Fig. 5C). Additionally, as recently shown by us [7] and others [32], [33], the activity of mTOR and S6 kinase are decreased following nutrient/growth factor deprivation and this relieves the inhibition of eEF-2 kinase [34]. Since protein synthesis devours ATP, termination of protein elongation via activation of eEF-2 kinase should conserve energy and support cell survival during times of starvation. Furthermore, cells depleted of eEF-2 kinase were unable to tolerate nutrient withdrawal as manifested by accelerated loss of viability when grown in serum-free media (Fig. 5), likely due to a critical depletion of ATP because of continued protein synthesis in the face of inadequate nutrients. This may also explain the increased sensitivity to growth factor inhibitors in tumor cells with silencing of eEF-2 kinase (Fig. 6, Table 1). MCF-7 cells are known to be deficient in caspase-3 and haplo-insufficient in *beclin 1* [35], [36]; however, in our study we found that the responses of MCF-7 cells to nutrient deprivation or growth factor inhibitors are not different from other breast cancer cell lines such as MDA-MB-468, suggesting that the effects of inhibiting eEF-2 kinase on autophagic response and cell survival/death are independent of caspase-3 and beclin 1.

In summary, the results reported here provide new evidence that activation of eEF-2 kinase and autophagy plays a protective role for breast cancer cells under metabolic stress including growth factor inhibition. By inhibiting autophagy, breast cancer cells should be less able to cope with nutrient/growth factor deprivation, resulting in better therapeutic outcomes for breast cancer patients. Thus, targeting the eEF-2 kinase-regulated autophagic survival may be exploited as a novel approach to preventing and overcoming refractoriness of cancer cells to growth factor inhibitors.

## Materials and Methods

### Reagents, antibodies and cell lines

Dulbecco's phosphate buffered saline (DPBS) and Hank's balanced salt solution (HBSS) were purchased from Invitrogen (Carlsbad, CA). E64, pepstatin A, protease inhibitor cocktail, phenylmethylsulfonyl fluoride (PMSF), 3-methyladenine (3-MA), 3-(4,5-dimethylthiazol-2-yl)-2,5-diphenyltetrazolium bromide (MTT), dimethyl sulfoxide (DMSO), trichloroacetic acid, anti-β-actin and anti-ran antibodies were purchased from Sigma-Aldrich (Saint Louis, MO). Anti-eEF-2 kinase, anti-phospho-eEF-2 kinase (S366), anti-eEF-2, and anti-phospho-eEF-2 (T56) antibodies were purchased from Cell Signaling Technologies (Beveley, MA). Anti-LC3B antibody was purchased from Novus Biologicals (Littleton, CO) and anti-BECN1 and anti-ATG5 antibodies were from Santa Cruz Biotechnology (Santa Cruz, CA). Anti-phospho-eEF-2 kinase (S398) antibody was a gift from Dr. Chistopher Proud (University of Dundee, UK). Human Breast cancer cell lines MCF-7 and MDA-MB-468 were obtained from American Type Culture Collection (ATCC, Manassa, VA). Cells were maintained in RPMI 1640 or DMEM medium supplemented with 10% fetal bovine serum at 37°C in a humidified incubator (5% CO_2_ and 95% air).

### siRNA and plasmid DNA transfection

For siRNA transfection, ON-TARGET plus siRNA oligos (Dharmacon, Lafayette, CO) and Oligofectamine (Invitrogen) were diluted with OPTI-MEM I reduced serum medium first, and then incubated for 20 minutes before adding to cell culture. Cells were transfected with siRNA for 24∼72 hours. The eEF-2 kinase siRNA (AAGCUCGAACCAGAAUGUCAA) was customer-designed and synthesized by Dharmacon. siRNA targeting human BECN1 (J-0105552-05) or human ATG5 (J-004374-07) and the non-targeting control siRNA (siCONTROL, D-001810-01) were purchased from Dharmacon. For plasmid DNA transfection, cells were tansfected with the mixture of plasmid pEGFP-LC3 and Lipofectamine 2000 (Invitrogen) in OPTI-MEM I reduced serum medium. After 6-hour incubation at 37°C, transfection medium was replaced with RPMI 1640 medium without antibiotics. GFP expression was detected under an inverted fluorescent microscope. Transfectants were selected with 600 µg/mL of G418 (Invitrogen) and maintained with 300 µg/ml of G418.

### Western blot analysis

Cells were washed twice with ice-cold PBS and scraped off the tissue culture dishes. Cells were collected in microcentrifuge tubes and centrifuged at 1000×g for 5 minutes. Cell pellets were lysed in ice-cold lysis buffer [20 mM Tris-HCl (pH 7.5), 150 mM NaCl, 1 mM EDTA, 1 mM EGTA, 1% Triton X-100, 2.5 mM sodium pyrophosphate, 1 mM ß-glycerolphosphate, 1 mM Na_3_VO_4_, 1x protease inhibitor cocktail, and 1 mM PMSF] and sonicated for 5 seconds. The lysates were clarified by centrifugation at 13,000×*g* for 15 minutes at 4°C. Thirty µg of proteins were resolved by SDS-PAGE and transferred to ImmunoBlot PVDF membrane (Bio-Rad Laboratories, Hercules, CA), and the membranes were probed with the respective antibodies. Pierce ECL Western Blotting Substrate kit (Pierce, Rockford, IL) was used to detect protein signals on the immuno-blots.

### Assay of protein synthesis

Protein synthesis rate was measured with a radio-labeling assay [37]. Briefly, cells seeded in 60-mm culture dishes were labeled with 25 µCi per ml of EasyTag EXPRESS ^35^S protein labeling mix (PerkinElmer, Boston, MA) in RPMI 1640 medium. After incubation at 37°C for 15 min, cells were washed 4 times with 4 ml of ice-cold PBS and then lysed in 200 µl of Complete Lysis-M lysis reagent with 1 Mini Protease Inhibitor Cocktail tablet per 10 ml of lysis reagent (Roche Diagnostics, Indianapolis, IN). Cell lysates were collected in a microfuge tube and clarified by centrifugation at 13000×g for 10 min at 4°C. Supernatant was precipitated with 20% of trichloroacetic acid and collected on GF/C filters (Millipore, Bedford, MA). Filters were washed 4 times with 1 ml of 10% trichloroacetic acid and subject to liquid scintillation counting. The specific activity of protein synthesis was determined by the amount of incorporated ^35^S-methionine/cysteine per mg of total protein per min.

### GFP-LC3 cleavage

Cells were transfected with a GFP-LC3-expressing plasmid. GFP-LC3-expressing cells were subjected to nutrient depletion in the presence of the lysosomal protease inhibitors (50 nM bafilomycin A1, 5 µg/ml E64D, 5 µg/ml leupeptin and 5 µg/ml pepstatin A). At the end of treatment, Cells were fixed with 4% formaldehyde in PBS (pH 7.4) for 15 min. To determine the autophagic response, cells are inspected at 60× magnification for total number of GFP-LC3 puncta. At least 150 cells are scored in each treatment [38].

### Measurement of cellular ATP

Cellular ATP was measured using a luminescence ATP detection assay system (ATPlite kit, PerkinElmer, Boston, MA). The assay was carried out in 96-well cell culture plates according to the manufacturer's instruction. The luminescence was detected on a PerkinElmer Victor 3 plate reader as counts per second (CPS). The relative ATP level was calculated by dividing the CPS of the treated samples by that of control samples.

### Cell viability assay

Cell viability was determined using MTT assay. Briefly, cells were seeded in 96-well tissue culture plates and subjected to different treatments. At the end of treatments, cells were incubated with 20 µl of 5 mg/ml MTT reagent. After 4-hour incubation at 37°C in a humidified atmosphere containing 5% CO_2_, formazan crystals were dissolved in 200 µl of DMSO. Absorbance at 570 nm was determined using a PerkinElmer Victor3 plate reader.
